# An Online Intervention to Promote Mental Health and Wellbeing for Young Adults Whose Parents Have Mental Illness and/or Substance Use Problems: Theoretical Basis and Intervention Description

**DOI:** 10.3389/fpsyt.2019.00059

**Published:** 2019-02-15

**Authors:** Andrea Reupert, Catherine Bartholomew, Rose Cuff, Kim Foster, Jodie Matar, Darryl J. Maybery, Laura Pettenuzzo

**Affiliations:** ^1^Faculty of Education, Krongold Clinic, Monash University Melbourne, VIC, Australia; ^2^Wellways Australia Incorporating Australian HealthCall Group Melbourne, VIC, Australia; ^3^The Bouverie Centre Melbourne, VIC, Australia; ^4^School of Nursing, Midwifery & Paramedicine, Australian Catholic University Melbourne, VIC, Australia; ^5^NorthWestern Mental Health Melbourne, VIC, Australia; ^6^Department of Rural Health, Monash University Clayton, VIC, Australia

**Keywords:** young adults, parents, substance use, mental illness, online intervention

## Abstract

The transition to adulthood can be a vulnerable period for certain population groups. In particular, young adults aged 18–25 years who have a parent with mental illness and/or substance use problems face increased risks to their mental health compared to same aged peers. Yet these young adults may not have access to age-appropriate, targeted interventions, nor engage with traditional face-to-face health services. To support this vulnerable group, services need to engage with them in environments where they are likely to seek help, such as the Internet. This paper describes the risk mechanisms for this group of young adults, and the theoretical and empirical basis, aims, features and content of a tailored online group intervention; mi.spot (**m**ental **i**llness: **s**upportive, **p**reventative, **o**nline, **t**argeted). The participatory approach employed to design the intervention is described. This involved working collaboratively with stakeholders (i.e., young adults, clinicians, researchers and website developers). Implementation considerations and future research priorities for an online approach targeting this group of young adults conclude the paper.

## Background

A key risk factor to young peoples' mental health and wellbeing is having a parent with a mental illness and/or substance use problem (1). The mechanisms underlying this association are accumulative and involve interactions between genetic, individual, parent, familial, environmental, and societal factors (1). Parental mental illness or substance use problems have been associated with various adverse outcomes for young people, including the development of their own mental illness or substance use problem, academic failure, high incarceration rates, and stress-related somatic health conditions such as asthma (2–5). These adverse impacts may be maintained into adulthood. The transition period from adolescence to adulthood is a critical time to intervene and attempt to reduce the onset of mental health problems (6). Given that 21–23% of children grow up with at least one parent with a mental illness (7) and 11.9% of children live with at least one parent who was dependent on or abused alcohol or an illicit drug (8), it is imperative that efforts are made to reduce the risk of intergenerational mental illness and substance misuse.

This article presents the theoretical and empirical basis for an online intervention, mi.spot (**m**ental **i**llness: **s**upportive, **p**reventative, **o**nline, **t**argeted) for young adults (aged 18–25 years) whose parents have a mental illness and/or substance use problem. First, the extant literature on the mechanisms by which risk is conferred on these young adults will be reviewed, followed by service gaps and young adults' preferences for support. This literature provides the foundation for a theoretical approach to the development of the online intervention, mi.spot. The approach employed to develop mi.spot is then described, followed by an outline of the intervention. The issues associated with introducing a new intervention such as mi.spot into regular service delivery, and opportunities for future research conclude the paper.

## Mechanisms of Risk Related to Mental Health Among Young Adults

Mental health disorders account for the highest burden of disease in adolescence and young adulthood. The majority of lifetime prevalence for mental health conditions occurs by 24 years of age, and substance abuse disorders by 25 years (6). According to Arnett (9), emerging adulthood is a distinct transitional period between adolescence and young adulthood (18–25 years) that marks a major transition in terms of identity, work, relationships, and place of residence. The changing roles and increasing levels of responsibility during this period increases stress (10) which may precipitate depressive episodes (11). Additionally, substance use problems and risky sexual practices increase during these ages (10). However, young adults have lower rates of mental illness treatment than adults or adolescents (12) and only 35% of young people experiencing mental health problems seek professional help (13) due to access issues and stigma (14). Although peer relationships are integral in this developmental phase, decreases in social support, which may occur as young people leave school, are linked with increases in depressive symptoms (15). During this transitional period emotional stability is not yet established, specifically dysregulation of anger, which is high compared to other age groups (16).

There are various mechanisms involved in the transmission of mental illness for young adults whose parents have a mental illness and/or substance use problem. The first potential risk mechanism is young adults' relationship with their parent and their need for independence. Marsh and Dickens (17) found that some young adults find it challenging to separate themselves from their families to pursue normative developmental tasks such as attending university. When they do attend university, these young adults describe greater psychosocial adjustment difficulties than their peers (18). Mitchell and Abraham (19) found that amongst university students, those with a parent with a mental illness experienced higher levels of homesickness compared to other young adults. Likewise, Bountress et al. (20) found that young adults whose parents had substance use problems were more likely to have negative experiences during the leaving home transition. In turn, this predicted an increased risk of affective disorders in adulthood. They suggested that parents with substance use problems may attempt to limit their children's independence from the family of origin or fail to appropriately scaffold their leaving home transition. Family dynamics can be strained. Abraham and Stein (21) found that young adults with a mother with a mental illness reported lower levels of affection from their mothers compared to their same aged peers who did not have a mother with a mental illness. Relatedly, parents with substance use problems have been found to be more emotionally withdrawn from their young adult children and to show less sensitivity and more hostility toward them, compared to those whose parents do not have substance use problems (20, 22).

Similarly, many young adults find it difficult to be emotionally independent from their parent who has a mental illness or substance use problem. Drawn from interviews with 12 young people aged 13–26 whose parents had substance use problems, Wangensteen et al. (23) described their struggle in balancing emotional closeness and distance with their parent. In particular, those aged 18–26 felt sorry for their parent and described a sense of obligation toward them and the subsequent struggle to separate themselves emotionally when they moved out of home. As some young adults whose parents have a mental illness find it difficult to be emotionally independent, they may find it challenging to regulate their emotions (24). Such research may explain, at least partially, why some young adults experience difficulty forming close personal or romantic relationships (3, 25). Kumar and Mattanah (26) found that early attachments provide a template for romantic and other relationship developments in early adulthood, and this appears to be particular pertinent for young adults whose parents have a mental illness or substance use problem.

Role reversal, parentification or caring responsibilities are other mechanisms of risk for this group of young adults (27). They may be given or assume the responsibility of caring for their parent and siblings and maintaining the household (25). These responsibilities may have adverse impacts on employment, schooling, and friendship groups (28, 29). Developmentally, stigma and a sense of shame associated with mental illness may increase as adolescents and young adults appreciate the manner in which others in the community perceive those with a mental illness, and so impede help-seeking for themselves and their family (30).

Whether children of parents with different mental health concerns have the same mechanisms of risk and subsequent intervention needs are questions that have been previously debated in the literature (31). In two systematic reviews of the impact of different parental mental illness on children's own diagnosis, van Santvoort et al. (32, 33) found that children of parents across the spectrum present with a broad-range of adverse outcomes, not limited to their parents' diagnosis. On this basis, the authors suggest that all children can be offered the same intervention, though simultaneously suggest that additional, tailored interventions might be required, such as might arise through the exposure to violence or abuse (33). Others have also argued that across diagnostic groups, young people may be exposed to similar familial and contextual stressors such as marital discord, housing instability, isolation, and poverty (27). Other common risks across parental diagnoses might include stigma, caring responsibilities, and a lack of accurate knowledge about their parent's illness (1, 27).

Young adults have highlighted a lack of knowledge about their genetic vulnerability to addiction and mental illness (34). While younger children are often left out of explanations about their parent's illness, Seurer (35) suggests that, for parental depression at least, some young adults may be viewed by their parents as confidantes even though these discussions do not always provide the full picture of what is happening for the parent. Knowing more about mental illness/substance use and in particular their parent's specific illness can help young adults understand and distance themselves from their parent (36, 37).

During the transition to adulthood, parental control declines while the influence of peers gain in importance (38). Similarly, young adults seek support and information from their peers, rather than parents (39). Giving opportunities to share experiences with peers is important when services want to connect with difficult to engage populations (40) and is also important for young people whose parents have a mental illness or substance use problem (41). Klodnick et al. (42) found that for young adults, associating with peers who have similar mental health experiences can provide strong affectional bonds, meaningful connections and useful exchanges about services. Emerging adulthood is perhaps the first time when individuals can formulate a “new and ideally integrative understanding of one's life story” and integrate “different personifications of the self within a single self-defining life story” (43). Thus, young adults may be actively involved in the communicative sense-making processes in coming to terms with their parent's illness or substance use and can articulate and share their life experiences with others (35), and if given the opportunity, with their peers (44).

In summary, the transition to adulthood can be a vulnerable period especially for those whose parents have mental illness and/or substance use problems. At the same time, emerging adulthood represents an ideal time to develop new strategies and foci in readiness for adulthood, in relation to key developmental tasks, such as identity formation, role transition, the formation of new social connections and intimate romantic attachments, and independence from parents. Moreover, promoting access to treatment services is necessary to improve mental health outcomes and reduce the burden of mental illness, especially those aimed at increasing young adults' willingness to seek help (45).

## Available Interventions and Service Gaps

There are various evidence-based interventions developed for children and young people aged under 18 years, who have a parent with a mental illness or substance use problem. These include peer support programs, family interventions and psychoeducational resources (46). In a systematic review and meta-analysis, Siegenhaler et al. (47) found that the risk of acquiring a parent's mental illness was reduced by 40% for children participating in targeted interventions. Most interventions for children and young people in these families draw on cognitive, behavioural, and/or psychosocial theories and deliver topics on mental health literacy, adaptive coping, and problem solving (48). However, the majority of interventions for children and young people in these families are limited to those aged under 18 years (48).

When asked about their preferred supports, adolescents aged 13–17 years and living in families where a parent has a mental illness or substance use problem, reported a clear preference for online supports (41). Extending that study, Matar et al. (44) employed a Delphi study with 282 young adults aged 16–21 years and whose parents had a mental illness and/or substance use problem and asked them what they wanted from an online intervention. Online opportunities to share with other young adults living in similar families was a common request as were topics on psycho-education, managing the parent-child relationship, and strategies to build resilience, and improve mental health, wellbeing, and coping. Finally, they wanted assurances that any online bullying would be dealt with appropriately.

There are some online interventions (mostly from the Netherlands) for young people whose parents have mental illness though these are still in the early stages of development and generally target both adolescent and young adults. These interventions include *Survivalkid* for 12–25 year olds (49, 50), *Grubbel* for 15–25 year olds (51) and *Kopstoring* for 16–25 year olds (52). To date, one randomised controlled trial evaluation has been completed on Kopstoring with positive trends found toward a reduction in internalising symptoms (52). No significant differences were found in self-reported depressive symptoms or internalising problems compared to treatment as usual in a 3-month follow-up, though the authors suggested this might be due to problems with the evaluation design (52). These interventions are based in Europe and developed specifically for those sociocultural and mental health service contexts. To date, there have been no reported interventions for this group of young adults from English-speaking countries, including Australia.

To succeed in identifying and supporting young adults who have a parent with a mental illness and/or substance use problem, services need to engage with young adults in environments where they seek help and interact. Wetterlin et al. (53) found that 61.6% of 521 young adults aged between 17 and 24 years had utilised the Internet to access information or seek help for how they were feeling, and 82.9% indicated that they were likely to use a mental health website to find information in challenging times. As young adults often prefer anonymous sources of help to traditional services (54), online interventions provide an ideal opportunity to intervene with this vulnerable group. Online approaches are important as they have the potential to link young adults with others in similar situations, especially those in rural and remote areas (55).

The present paper is the first to provide a theoretical overview and empirical basis for an online intervention for this particular group of vulnerable young adults from an English-speaking country. Indeed, theoretical frameworks for online interventions are often missing (56) but critical for ongoing intervention monitoring and evaluation (57).

## mi.spot: Developing an Online Approach

The mi.spot intervention was developed iteratively over a 36 month period using participatory design principles and drawing on relevant theory, existing research and feedback from feasibility trials (see Table 1).

**Table 1:** How mi.spot was developed.

**Sources**	**References**
Reference group using participatory design principles Theory:	(56, 57)
• Competence enhancement model	(58–60)
• Theory of health information seeking behaviour	(61)
• Theory of development: emerging adulthood	(9, 10)
Previous research on parental mental illness and substance use problems in particular	
• Common intervention ingredients	(46)
• Adolescents' preferences for support	(39)
• Young adults' preferences for online support	(42)
Elements of effective online interventions for young people	(51, 62, 63)
Four feasibility trials conducted, with feedback elicited from 66 participants and five facilitators	(64)

From the outset, a *participatory design approach* was employed (58, 59) through the creation and facilitation of a reference group consisting of researchers, clinicians, website designers, and young adults who have lived experience of parents with mental illness and/or substance use problems. The group was collaborative and collegiate with all stakeholders recognised as having different but equally important skills to offer. In particular, young adults were included to ensure that the intervention was user friendly and acceptable and clinicians were involved as the intervention needed to be clinically feasible. It was acknowledged that what young people want can be different from what parents and clinicians believe they need (60), which meant privileging the views of the young people on the reference group, especially in regard to site aesthetics and content and in terms of how clinicians might respond to young people's questions and concerns. Regular face-to-face meetings were facilitated with the aim of raising a diversity of views and learning from each other. Different members of the group had worked together previously (on other research projects, or on face-to-face peer support programs) which provided opportunities for capacity building. The reference group met regularly over 36 months to discuss website developments and the implications of research (see below) to the website. Notes were taken at each meeting to document decisions and follow-up meetings with individuals were conducted as required to ensure that all perspectives were considered. Overall, the participatory design approach covered several phases; the identification of problems for young people living these families (drawing on existing research, see below) the generation of solutions, the development of and feedback on an online intervention, and the development of an implementation and evaluation strategy, including dissemination procedures.

*Theoretically*, mi.spot is based on the competence enhancement model, discussed in detail by Barry (65). The model takes a lifespan approach to the promotion of mental health, and focuses on enhancing strengths and promoting resilience (61, 65). According to Eccles and Appleton (62), the model is most beneficial when there are explicit efforts to promote connections to others and enhance participants' competence in developmentally appropriate domains. Cognitive and behavioural approaches are commonly incorporated within the competence enhancement model (62), given their effectiveness and efficacy in improving dysfunctional cognition, regulating emotions and the promotion of adaptive problem solving (63). The theory of development for young adults was used for the current intervention, with a focus on identify formation, managing relationships and independence (9, 10). Additionally, the theory of health information seeking behaviour (64) was applied to promote optimal use of the intervention. This approach acknowledges the role of passive and active retrieval of information, and the role of peers and clinicians in helping to process and interpret information (64).

In terms of *research*, the intervention drew on previous work that identified the ingredients commonly associated with effective interventions for this target group (48). Previous research that sought the views of young adults whose parents have mental illness and/or substance use problems regarding support in general (41), and online interventions in particular (44), were incorporated. Evidence on online behaviour change interventions was used to inform online functionality, especially those designed for adolescents and young adults (53). A systematic review found that effective online interventions provides opportunities for interaction, personalized and normative feedback, and self-monitoring (66). Likewise, Short et al., (67) drew on user engagement research across multiple disciplines to identify those factors that influence how users engage with online interventions. In their model, engagement is influenced by (i) the environment including the length of time available to the user and the user's access to the Internet (ii) individual factors related to perceived personal relevance and usefulness of the intervention and their expectations and (iii) intervention design including the interactivity, aesthetics, credibility and opportunities to interact with a counsellor. These features were considered in the development of mi.spot.

*Seven feasibility trials* of mi.spot have been conducted since its inception, with 66 participants. According to Eldridge et al. (68) feasibility trials ascertain whether an intervention can be done, whether it should be done and if so, how. Related issues that feasibility studies may address include participants' willingness to be recruited, the time required to collect and analyze data, and the acceptability and suitability of any given intervention for both clinicians and participants. Various methods were used to recruit participants for mi.spot with Facebook advertisements found to be the most effective and efficient. Feedback from previous participants and online facilitators in semi-structured interviews were used to modify the initial iteration of the intervention. For example, many previous participants wanted to see a greater emphasis on managing friendship, work, and significant relationships and not only managing relationships with their parent and these additions were subsequently incorporated; facilitators requested additional functions such as notification of someone typing (i.e., “…”) in the online group chats to allow for more effective facilitation.

## mi.spot: the Intervention

mi.spot is an online, 6 week voluntary intervention for groups of up to 20 young adults (aged 18–25), who have a parent with a mental illness and/or substance use problem. mi.spot aims to (i) improve knowledge about mental health and wellbeing, (ii) promote adaptive coping, (iii) build, expand, and sustain healthy relationships, (iv) increase resilience, (v) encourage help seeking behaviour, (vi) facilitate a sense of peer connection and finally (vii) foster mental health and wellbeing. mi.spot is a manualised approach that offers real time (synchronous) and anonymous peer online networking opportunities and an individually tailored, interactive and professionally led intervention, as evident by the following functions:

- Six, one hour, professionally facilitated psychoeducational modules delivered online in synchronous mode (see Table 2).
- A private, online diary (called mi.thoughts.spot) to prompt and encourage participants to apply a cognitive behavioural approach to current stressful situations, with the support of a facilitator (provided asynchronously).
- Opportunities for participants to chat informally with each other on threads (i.e., topics) initiated by a participant or facilitator.
- Opportunities for one-to-one private online counselling sessions between a participant and a facilitator (synchronous).
- Video, audio, print resources, and self-monitoring questionnaires offered as weekly activities for participants in order to consolidate and extend learning from weekly sessions.

**Table 2:** mi.spot weekly modules.

**Module**	**Module description**
Week 1: What's mi.spot all about?	This week provides an orientation to the intervention and various cognitive-based strategies to help participants identify unhelpful thinking patterns, reframe these in a more constructive manner, and regulate emotions. Time is spent practising these strategies.
Week 2: Learning about mental health and mental illness	Participants are offered the opportunity to ask questions about mental health and illness, with a particular focus on their parent's illness, or substance use problem. They are encouraged to discuss how their parents' illness or substance use problem may impact their own health and wellbeing. Information about generic vulnerabilities is provided with an emphasis on ways to promote mental health and wellbeing.
Week 3: Me, my parent and other relationships	Participants are encouraged to reflect on their current relationship with their parent, and how the dynamics of the parent-child relationship might influence other relationships. Strategies for healthy boundary setting are discussed.
Week 4: Managing stress	Participants are invited to identify current stressor/s and as a group, discuss adaptive coping strategies.
Week 5: Caring—who, me?	Participants are asked to identify ways they might look after themselves (i.e., physically and emotionally), the barriers to self-care, and how these might be overcome. Their caring responsibilities are discussed and integrated with the prior module on boundary setting in relationships.
Week 6: Taking control of my life	The final module consists of a general overview of key aspects of previous modules. It emphasizes strengths, provides recommendations for self-care, and encourages help seeking and the continued use of adaptive appraisal strategies.

Participants may elect to join all, some or none of the offerings. The password protected site is moderated twice daily including weekends. Procedures exist for rule violations (e.g., online bullying) and risk procedures (e.g., if a participant expresses suicidal thoughts).

## Online Facilitator Role and Training

Online facilitators for mi.spot assume an active role in encouraging ongoing, supportive conversations between participants, facilitating the weekly sessions, and providing one to one online counselling sessions as required. They also fulfil a monitoring role and ensure that the site is a safe, respectful space for everyone by identifying and addressing any instances of bullying, harassment, and racism and/or descriptions of harm to self or others.

Currently, facilitators are master's level psychology students working in a university clinic but any qualified mental health clinician would be able to deliver the intervention after appropriate training had been attended. Two days of facilitator training using manualised content are provided. The first day focuses on generic online counselling skills (in both group and individual counselling mode) and the second specifically examines the mi.spot intervention and how it should be delivered. The second day starts with a presentation from two young adults with lived experience who discuss their experiences and preferences for online interactions. The remaining part of the day is spent on simulated training scenarios to practice responses on the site. Fidelity checks for the weekly sessions are built into the intervention at the facilitator level, to ensure all topics are covered. Future considerations are to deliver the training online [as per (69)].

## Future Priorities

Building on the current evidence base in this area (52), further randomised controlled trials are needed to establish the effectiveness and efficacy of mi.spot, including cost effectiveness. Comparing the online intervention, mi.spot, with face-to-face peer support programs will be important to evaluate comparative effectiveness. Uptake, engagement and drop out numbers as well as website analytics need to be documented to establish which participants engage with the different components of the intervention and the impact that this may have on participant outcomes. Monitoring of the intervention is required to ascertain to what extent the intervention been implemented as designed (as per the manual) and what modifications might be required. Safety is another evaluation component which may be measured through clinical deterioration related to intervention use and inappropriate use (e.g., online bullying). Developing online interventions for young people aged 13 to 18 years living in these families is another priority.

Further information is needed on how mi.spot may be embedded into services. Batterham et al. (70) identified various barriers for delivering online interventions including policy, safety, and political restrictions, incompatible reimbursement systems, limited availability of trained clinicians, and clinicians' negative attitudes toward Internet interventions. Nonetheless, a recent study found that Australian clinicians were highly supportive of online interventions for this particular group of young people as they acknowledged that many miss out on face-to-face peer support programs (71). Most research on online interventions has focused on efficacy and effectiveness trials, with little investigation on implementation processes (72), making this a priority for future research. Within these deliberations, the applicability and effectiveness of a stepped care model needs to be investigated, where mi.spot may be provided as a first step in intervention, or alternatively delivered in conjunction with individual face-to-face support. Relatedly, a strategy for targeting young people is needed. Building on the successful method of recruiting participants in the feasibility trials using Facebook advertising, other potential approaches include Twitter and YouTube advertisements and by promoting the intervention via various university and community mental health agencies. Young people's input into these approaches will be critical.

At present mi.spot is delivered from a university clinic but our vision is to expand the service into mainstream settings. Which service or services might assume this responsibility is being considered. Child and adolescent mental health services might appear to be an appropriate service but traditionally focus on those aged under 18 years. Others have highlighted the problems transitioning adolescents into the adult mental health system as well as the inappropriateness of adult mental health services for young adults (73). This is concerning, as service inadequacies and gaps during the transitional period from adolescence to adulthood have the potential for long lasting functional difficulties (74). Conversely, compared to standard adult services, age-specific interventions may increase young adults' use of mental health services (75). Given the vulnerability, prevalence, and the unique needs of young adults whose parents have a mental illness or substance use problem, it is crucial that developmentally informed and relevant services and interventions are developed and, arguably more importantly, delivered on a broad scale.

## Author Contributions

AR, CB, RC, KF, JM, DM, and LP contributed to the conception of the paper. AR wrote the first draft of the manuscript. RC, KF, JM, DM, and LP wrote sections of the manuscript. All authors contributed to manuscript revision, read and approved the submitted version.

### Conflict of Interest Statement

The authors declare that the research was conducted in the absence of any commercial or financial relationships that could be construed as a potential conflict of interest.
